# Inhibitory Effect of TNF-α on Malaria Pre-Erythrocytic Stage Development: Influence of Host Hepatocyte/Parasite Combinations

**DOI:** 10.1371/journal.pone.0017464

**Published:** 2011-03-04

**Authors:** Nadya Depinay, Jean Francois Franetich, Anne Charlotte Grüner, Marjorie Mauduit, Jean-Marc Chavatte, Adrian J. F. Luty, Geert-Jan van Gemert, Robert W. Sauerwein, Jean-Michel Siksik, Laurent Hannoun, Dominique Mazier, Georges Snounou, Laurent Rénia

**Affiliations:** 1 Institut Cochin, Département d'Immunologie, Université Paris Descartes, CNRS (UMR 8104), Paris, France; 2 INSERM, U567, Paris, France; 3 INSERM UMRS 945, Paris, France; 4 Université Pierre & Marie Curie, Faculté de Médecine Pitié-Salpêtrière, Paris, France; 5 Singapore Immunology Network, Agency for Science, Technology and Research (A*STAR), Biopolis, Singapore, Singapore; 6 Equipe Parasitologie Comparée et Modèles Expérimentaux USM0307, CNRS IFR101, Muséum National d'Histoire Naturelle, Paris, France; 7 Department of Medical Microbiology, Radboud University Nijmegen Medical Centre, Nijmegen, The Netherlands; 8 Assistance Publique-Hôpitaux de Paris, Service de Chirurgie Digestive, Hépato-Bilio-Pancréatique et Transplantation Hépatique, Centre Hospitalo-Universitaire Pitié-Salpêtrière, Paris, France; 9 AP HP, Centre Hospitalo-Universitaire Pitié-Salpêtrière, Paris, France; State University of Campinas, Brazil

## Abstract

**Background:**

The liver stages of malaria parasites are inhibited by cytokines such as interferon-γ or Interleukin (IL)-6. Binding of these cytokines to their receptors at the surface of the infected hepatocytes leads to the production of nitric oxide (NO) and radical oxygen intermediates (ROI), which kill hepatic parasites. However, conflicting results were obtained with TNF-α possibly because of differences in the models used. We have reassessed the role of TNF-α in the different cellular systems used to study the *Plasmodium* pre-erythrocytic stages.

**Methods and Findings:**

Human or mouse TNF-α were tested against human and rodent malaria parasites grown *in vitro* in human or rodent primary hepatocytes, or in hepatoma cell lines. Our data demonstrated that TNF-α treatment prevents the development of malaria pre-erythrocytic stages. This inhibitory effect however varies with the infecting parasite species and with the nature and origin of the cytokine and hepatocytes. Inhibition was only observed for all parasite species tested when hepatocytes were pre-incubated 24 or 48 hrs before infection and activity was directed only against early hepatic parasite. We further showed that TNF-α inhibition was mediated by a soluble factor present in the supernatant of TNF-α stimulated hepatocytes but it was not related to NO or ROI. Treatment TNF-α prevents the development of human and rodent malaria pre-erythrocytic stages through the activity of a mediator that remains to be identified.

**Conclusions:**

Treatment TNF-α prevents the development of human and rodent malaria pre-erythrocytic stages through the activity of a mediator that remains to be identified. However, the nature of the cytokine-host cell-parasite combination must be carefully considered for extrapolation to the human infection.

## Introduction

Tumour necrosis factor (TNF)-α is a cytokine with pleiotropic effects including anti-microbial activities [1]. In malaria infections, it has been shown that TNF-α could have both beneficial and detrimental effects. TNF-α is detected in the circulation during the erythrocytic phase of the infection in humans [1]–[3] and in mice [4], [5]). In both hosts, high levels of this cytokine have been associated with malarial pathology such as fever [6], and cerebral malaria [3], [5]. On the other hand, TNF-α has also been shown to have a potent anti-parasitic activity. Administration of recombinant TNF-α protected against blood stage infection with *Plasmodium chabaudi* in mice [7], while mice deficient for TNF-α controlled *P. chabaudi adami* blood infections less efficiently [8]. In humans, sustained high levels of TNF-α were associated with rapid clearance of fever and parasites [9]. There are controversial observations concerning the role of TNF-α against the pre-erythrocytic (PE) stages of the malarial infection. Schofield *et al*. reported that recombinant human TNF-α directly inhibited *P. berghei* grown *in vitro* in the HepG2 human hepatoma cell line, and *in vivo* in rats and mice [10]. However, we have previously found that *P. yoelii* parasites grown in cultured purified mouse primary hepatocytes were unaffected by TNF-α, whereas this cytokine inhibited the hepatic development of this parasite *in vivo* [11]. We then showed for this rodent malaria model that the inhibitory effect observed *in vivo* was actually mediated by the IL-6 secreted by non-parenchymal liver cells in response to TNF-α stimulation [11]. Thus depending on the host/parasite combination, different effects of TNF-α on PE parasites were reported. In this study, we wished to reassess the role of some of the biological and experimental parameters on the inhibition of *Plasmodium* hepatic stages observed in *in vitro* assays of TNF-α activity.

## Materials and Methods

### Ethics

All experiments and procedures involving mice were approved by the “Direction Départementale des Service Vétérinaires de Paris, France” (Authorisation No 75–129) and performed in compliance with regulations of the French Ministry of Agriculture for animal experimentation (1987). Human liver fragments used to prepare primary hepatocyte cultures were collected after written informed consent from patients undergoing a partial hepatectomy. The collection and use of these tissues were undertaken in accordance with French national ethical regulations and have been approved by the Ethic Committee of the Centre Hospitalo-Universitaire Pitié-Salpêtrière, Assistance Publique-Hôpitaux de Paris, Paris, France.

### Cytokines and chemicals

Different batches of human and murine TNF-α with similar specific activity were obtained from R&D systems. Endotoxin levels in the different batches used in this study were always below 1.0 EU per 1 µg as reported by the manufacturer. S-methyl-thiourea (SMT, Sigma), a potent inhibitor of iNOS [12] and N-acetyl-cysteine (NAC), which prevents oxygen free radical [13], were obtained from Sigma.

### Mice and parasites

BALB/cJ mice were purchased from Harlan Laboratories (Gannat, France), and were housed in a pathogen-free rodent barrier facility. Sporozoites of the uncloned line of *Plasmodium yoelii yoelii* 265BY strain (Pyy265BY), of *P. yoelii yoelii* 17X strain clone 1.1 (Py17X), and of *P. berghei* ANKA cloned line transfected with GFP (PbA) [14] were obtained from infected salivary glands of *Anopheles stephensi* mosquitoes, 16 to 21 days after an infective mouse blood meal. After aseptic dissection, salivary glands were homogenized in a glass grinder and diluted in culture medium [15]. Sporozoites of the NF54 strain of *Plasmodium falciparum* were obtained from infected salivary glands of *A. gambiae* mosquitoes which have been fed two weeks before on infected human blood cultures using a membrane-based feeder system [16]. After aseptic dissection, salivary glands were disrupted by trituration in a glass tissue grinder, diluted in culture medium and the sporozoites were counted using a KovaSlide® chamber.

### Cells

Mouse hepatoma cells Hepa1–6 (ATCC CRL-1830) (10^5^ per well), were cultured in DMEM (Invitrogen) supplemented with 10% FCS (Invitrogen), 2 mM glutamine, 1% penicillin-streptomycin of a 100 X stock solution (Invitrogen). Human hepatocarcinoma cells HepG2/CD81+ (HepG2 stably expressing CD81) (10^5^ per well) [17] were cultured in supplemented DMEM as above, in culture dishes coated with rat tail collagen I (Becton Dickinson. Mouse primary hepatocytes were prepared as described, with minor modifications [18]. Cells were isolated by collagenase perfusion (Boehringer Mannheim) of liver fragments and were further purified over a 60% Percoll gradient (Pharmacia Biotech, Uppsala, Sweden). Mouse hepatocyte purity and viability were >95% as assessed by Trypan blue dye exclusion. Human primary hepatocyte cultures were prepared as described with minor modifications [19]. Briefly, cells were isolated by collagenase (Roche) perfusion of human liver fragments, which were collected and used in agreement with the French ethical regulations, and were further purified over a 40% Percoll gradient. Human hepatocyte purity and viability were >99% as assessed by Trypan blue dye exclusion. Mouse and human hepatocytes were seeded in eight-chamber plastic Lab-Tek slides (Nunc) coated with rat tail collagen I (Becton Dickinson) at a density of 1×10^5^ cells per well for primary murine hepatocytes, and of 2×10^5^ cells per well for primary human hepatocytes cells, and cultured at 37°C, 5% CO_2_, in DMEM medium as above supplemented with 10^−7^ M dexamethasone (Sigma) after complete cell adherence (12–24 hours).

### Evaluation of TNF-α cytotoxicity

Toxicity of the cytokine to primary culture of hepatocytes or hepatoma cell lines in flat-bottom 96 wells (20×10^3^ cells/well) was evaluated using a methylthiazolyldiphenyl-tetrazolium bromide (MTT) assay. Briefly, cytokines were added in triplicate at decreasing concentrations. Medium was replaced 3 hours after sporozoite infection and every day thereafter up to day 4 (for rodent parasites) or 7 (for *P. falciparum*) with fresh medium containing the cytokine at the same concentration. Twenty-four hours after the last medium change, 110 µl of a solution containing 10 µl of MTT (Sigma) solution (5 mg/ml) and 100 µl of medium were added and the cultures incubated for a further 4 hours. The formazan crystals that were formed were dissolved using 100 µl of a 1∶1 DMSO: ethanol solution. Optical density was read immediately at 570 nm with a reference wavelength at 630 nm [20].

### *In vitro* assay of sporozoite invasion of and development in hepatocytes and HepG2 cells

After removal of medium from the culture chambers, ten thousands sporozoites were added in 100 µl of fresh supplemented medium with various quantities of TNF-α tested, and at different times during cultivation. Medium was replaced at −24, 0, 3, and 24 h after rodent malaria sporozoite inoculation, and at −24, 0, 3, 24 h and then every day up to day 5 after *P. falciparum* sporozoite inoculation of primary human hepatocytes with fresh medium containing or not the cytokine. In one experiment, supernatant was collected from wells containing human hepatocytes treated with TNF-α (100 ng/m) of for 2 days. *Plasmodium* sporozoites (10^4^ in a 50 µl) were added to this supernatant (50 µl) or to fresh medium (50 µl). The final solution (100 µl) containing the sporozoite was added to the culture. Medium was replaced after 3 h, 24 h, and then every day up to day 5 after sporozoite inoculation. Experimental determination of the number of liver stage parasites was performed in triplicate or quadruplicate. Cultures were stopped 48 h (for rodent malaria species) or 5 days (for *P. falciparum*) after sporozoite infection, fixed with cold methanol and schizont numbers were assessed by immunofluorescence using antibodies recognizing *Plasmodium* liver stages as previously described [21], [22] and were quantified by microscopic examination or using the Odyssey infra red imaging system (Li-COR Biosciences) [23]. Percentage of inhibition of the development is calculated by comparing the numbers of parasitic forms in the experimental wells versus control wells.

## Results

### Effect of human TNF-α on HepG2-CD81 cells infected with murine *Plasmodium* species

HepG2 is a hepatoma cell line easily propagated *in vitro* [24] that has been shown to sustain the development of the *P. berghei* [25] but not the *P. yoelii* [26] liver stages. Recently Silvie *et al*. [17] showed that HepG2 cells transduced with CD81 are thereby made permissive to *P. yoelii* development, providing a good system to study *P. yoelii* liver stage biology, thus reducing the need for the more tedious primary murine hepatocyte cultures. We employed this cell line to assess the role of TNF-α against the hepatic stages of malaria parasites.

**Figure 1:**
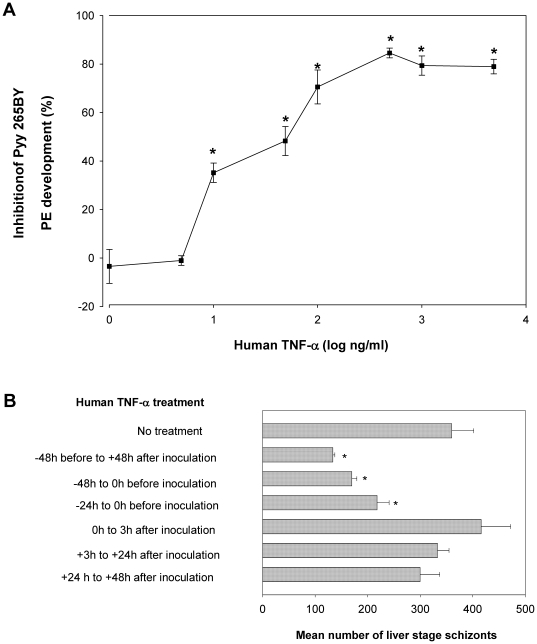
Human TNF-α inhibits the pre-erythrocytic stage of Pyy265BY. A. HepG2/CD81 hepatoma cell cultures were treated with various concentrations of recombinant human TNF-α, 48 h prior to, at the time of, and then 3 h and 24 h after sporozoite inoculation. Cultures were stopped 45 h after infection. Data are presented as the mean (± SD) reduction in Pyy265 liver schizonts numbers and represent data from two independent experiments (one represented by filled circles and the other by open circles). Parasite number reduction was calculated by enumerating 48 h liver schizonts in triplicate cultures exposed or not to TNF-α. Data are presented as the mean (± SD) reduction in liver schizont numbers in triplicate wells compared to the mean number in 6 control wells. The number of liver schizonts in control wells was 67±9.9. B. Timing of the antiparasitic effect of human TNF-α. HepG2/CD81 cells were incubated with 100 ng/ml of human TNF-α for different times. Cultures were stopped 48 h after sporozoite inoculation. Data are presented as the mean (± SD) numbers of liver schizonts in triplicate experimental wells and in six control wells. * *p*<0.05 versus control non-treated cultures (Kruskal-Wallis test, followed by Dunn test). The rate of infection Pyy265 sporozoites for HepG2/CD81 hepatoma cell was between 0.5 to 2% depending on the experiment. Data are representative of three independent experiments with similar results.

When human TNF-α was added to HepG2-CD81 cultures over a four-day period centred on the time of sporozoite inoculation, Pyy265BY hepatic parasite development was inhibited in a dose dependent manner (Figure 1A). Significant inhibition was observed even at a low dose of 10 ng/ml. Only minor increases in the level of inhibition were obtained at doses above 100 ng/ml (Figure 1A). We then tested the influence of the timing of TNF-α addition on the level of inhibition observed. HepG2-CD81 cultures where the cytokine was added only 1 day before sporozoite inoculation were also significantly inhibited by 100 ng/ml of TNF-α (Figure 1B). However, no inhibition of parasite development could be observed when TNF-α was first added starting at the time of sporozoite inoculation or thereafter (Figure 1B). The inhibitory activity of TNF-α was specific to the parasites because TNF-α had no cytotoxic effect on infected or normal HepG2 cells as measured by the MTT assay, even when added at the highest doses used. Indeed, the optical density values obtained in wells containing untreated cells [(0.953±0.2 arbitrary units (A.U.)] or cells treated for 4 days with 100 ng/ml TNF-α and infected with *P. yoelii* sporozoites (1.29±0.0.05 A.U.) did not differ significantly.

**Figure 2:**
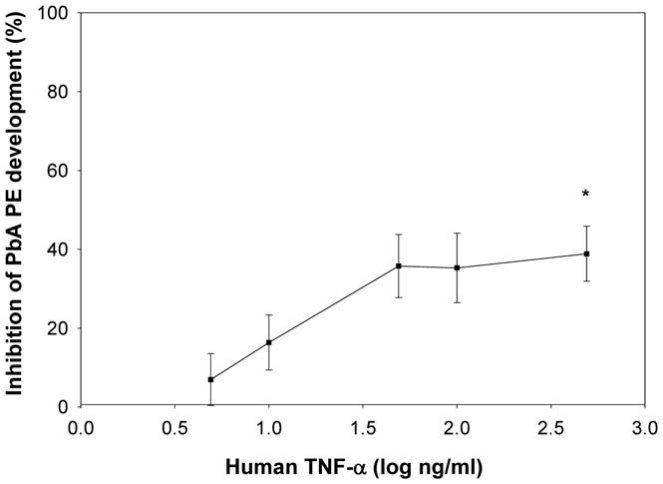
Human TNF-α inhibits the pre-erythrocytic stage of PbA. HepG2/CD81 hepatoma cell cultures were treated with various concentrations of recombinant human TNF-α, 48 h prior to, at the time of, and then 3 h and 24 h after sporozoite inoculation. Cultures were stopped 48 h after sporozoite inoculation. Data are presented as the mean (± SD) reduction in 48 h PbA liver schizont numbers from triplicate experimental wells compared to those from 8 control wells. The number of liver schizonts in the control wells was 357±31. * *p*<0.05 versus control non-treated cultures (Kruskal-Wallis test, followed by Dunn test). The rate of infection PbA sporozoites for HepG2/CD81 hepatoma cell was between 0.3 to 2% depending on the experiment. Data are representative of two independent experiments with similar results.

We next tested the effect of human TNF-α on PbA parasites, thus providing a reasonable comparison with the study of Schofield *et al.* where an inhibitory effect of human TNF-α against *P. berghei* NK65 strain was reported [10]. When human TNF-α at an inhibiting dose of 100 ng/ml was added to HepG2-CD81 cultures starting 48 h before sporozoite inoculation and over a four-day period until the end of the parasite cultivation period 48 h thereafter, PbA liver stage development was also inhibited in a dose dependent manner (Figure 2), though the magnitude of the inhibition was lesser than that observed for Pyy265BY.

### Effect of TNF-α on mouse hepatoma cell line Hepa 1–6 and primary mouse hepatocytes infected with murine *Plasmodium* species

**Figure 3:**
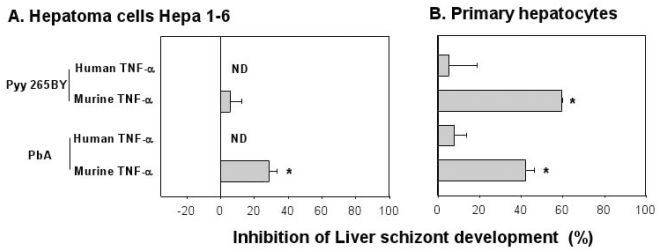
Effect of human or mouse TNF-α against the pre-erythrocytic stage of PbA and Pyy265BY grown in Hepa 1–6 hepatoma cells or primary hepatocytes cultures. (A) Hepa 1–6 cells and (B) primary hepatocyte cultures were treated with 100 ng/ml of recombinant human or mouse TNF-α, 48 h prior to, at the time of, and then 3 h and 24 h after sporozoite inoculation. Cultures were stopped 48 h later. Data are presented as the mean (± SD) reduction in PbA liver schizont numbers at 48 h in triplicate experimental wells as compared to those enumerated in 6 control wells. In the control wells, there were 110.3±13.1 liver schizonts in Hepa1-6 and 602±83.4 in the primary mouse hepatocytes infected with PbA, and 25.5±3.6 liver schizonts in Hepa1-6 and 443.5±133.5 in primary hepatocytes infected with Pyy265 BY. * *p*<0.05 versus control non-treated cultures (Kruskal-Wallis test, followed by Dunn test). The rate of infection for Pyy265 sporozoites was 0.02-.004% for Hepa1.6 cells and 0.1–2% for primary mouse hepatocytes. For PbA sporozoites, it was of 0.1–0.5% for Hepa1.6 cells and 0.5–1% for primary mouse hepatocytes. Data are representative of two independent experiments with similar results. ND, not done.

In order to test whether the findings obtained using HepG2-CD81 cells can be extended to other host cell/parasite combinations, we repeated the TNF-α treatment (100 ng/ml initiated at 48 h before sporozoite inoculation and maintained for the subsequent 48 h) with mouse hepatoma cells Hepa 1–6 and primary cultures of purified mouse hepatocytes, both of which sustain the growth of *Plasmodium* species that infect rodents [18], [27]. Mouse TNFα significantly inhibited PbA in Hepa1-6 cells (Figure 3A) or in primary hepatocyte cultures (Figure 3B), whereas for Pyy265BY, inhibition was only observed in primary hepatocytes (Figure 3).

We next tested the effect of human TNF-α on mouse cells since it was shown previously that *in vivo* treatment with this cytokine inhibited *P. berghei* NK65 liver stage development [10]. However, human TNF-α may have an indirect inhibitory effect on the liver stage *in situ*, for e.g. by inducing the production of another anti-liver stage cytokine such as IL-6 by nonparenchymal liver cells. We wished to ascertain whether the inhibition noted above was due to direct interaction with hepatocytes [11]. The liver stage development of parasites (Pyy265BY or PbA) grown in primary mouse hepatocytes was not inhibited by treatment with human TNF-α (Figure 3B). These results indicate that parasite species are differentially susceptible to the inhibitory activity of TNF-α, and that this is influenced by the origin of the TNF-α as well as the type and origin of the host cells in which the parasites develop. It is worth noting that treatment with TNF-α had no cytotoxic effect on infected or non-infected primary mouse cells as measured by the MTT assay. The difference in the optical density values obtained for wells containing untreated cells (0.47±0.09 A.U.) or cells treated for 4 days with 100 ng/ml TNF-α and infected with *P. yoelii* sporozoites (0.41±0.05 A.U.) were not significant.

### Effect of TNF-α on primary human hepatocytes infected with *P. falciparum*

**Figure 4:**
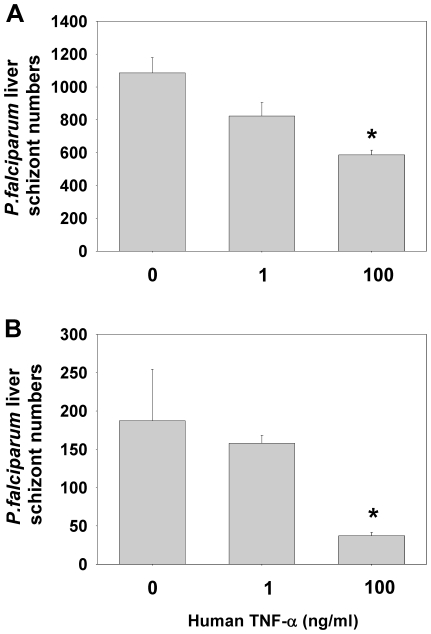
Human TNF-α inhibits the pre-erythrocytic stage of *P. falciparum*. Primary human hepatocyte cell cultures were treated with various concentrations of recombinant human TNF-α, 24 h (A) or 48 h (B) before, at the time of sporozoite inoculation, and then every day for day 1 to day 5. Cultures were stopped 5 days later after sporozoite inoculation. The data presented are mean numbers (± SD) of 5 days *P. falciparum* liver stages from triplicate experimental wells and from 8 control wells. * *p*<0.05 versus control non-treated cultures (Kruskal-Wallis test, followed by Dunn test). The rate of infection for *P. falciparum* sporozoites was 0.2–0.5% for primary human hepatocytes depending on the experiment. Data are representative of two independent experiments with similar results.

We then tested the effect of TNF-α on the development of *P. falciparum* in human primary hepatocytes, a host/parasite combination of direct clinical relevance. We used highly purified human hepatocytes to prevent indirect effect of TNF-α on contaminating nonparenchymal cells as shown previously [11]. *P. falciparum* liver stage development was inhibited when the cultures were treated with human TNF-α at 100 ng/ml, but not 1 ng/ml, 24 h or 48 h prior to sporozoite inoculation and until day 5 thereafter (Figure 4). Inhibition was more pronounced when the hepatocytes were pre-incubated with human TNF-α 48 h as compared to 24 h before sporozoite inoculation. As for the other hepatocyte cells, treatment with TNF-α had no cytotoxic effect on primary human hepatocytes as measured by the MTT assay. The optical density values obtained for wells containing untreated cells (0.78±0.005 A.U.) or cells treated with for 4 days with 100 ng/ml TNF-α and infected with *P. yoelii* sporozoites (0.793±0.03 A.U.) did not differ significantly.

### Absence of effect of nitric oxide derivatives and radical oxygen intermediates inhibitors on TNF-α mediated pre-erythrocytic stage inhibition

**Figure 5:**
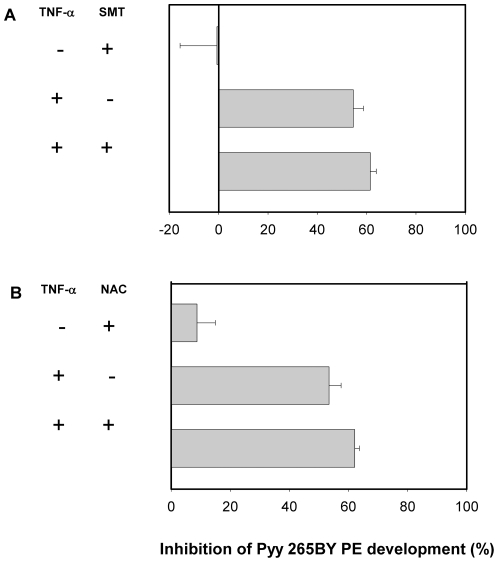
The effect of human TNF-α against Pyy265BY is not mediated by NO or ROI. HepG2/CD81 hepatoma cells treated or not with 100 ng/ml of human TNF-α together with or without SMT (A) or NAC (B) 48 h before, at the time of, and then 3 h and 24 h after sporozoite inoculation. Cultures were stopped 48 h later. Data are presented as the mean (± SD) reduction in liver schizont numbers in triplicate wells compared to the mean number in 6 control wells. The numbers of Pyy265BY liver schizonts in the 6 control wells were 145.5±9 (A), and 148±9 (B). The results are representative of three independent experiments. * *p*<0.05 versus control non-treated cultures (Kruskal-Wallis test, followed by Dunn test).

**Figure 6:**
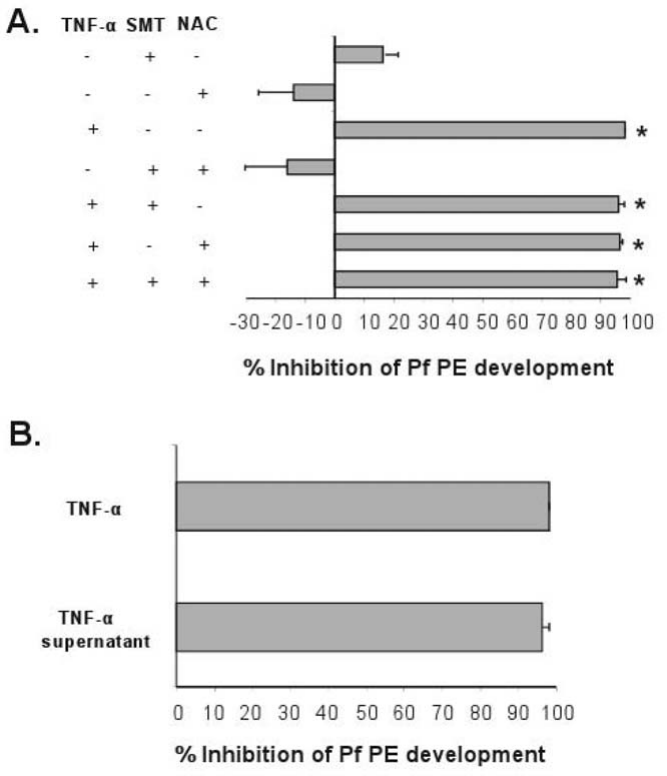
A soluble mediator but not NO or RO intermediates synthesized by human TNF-α-stimulated human hepatocytes inhibits *P. falciparum* development. A. Primary human hepatocytes were treated or not with 100 ng/ml of human TNF-α together with or without SMT or NAC at 48 h before, at the time and every day for day 1 to day 5 after sporozoite inoculation. B. In the same experiment, supernatants from cells treated previously for 48 h with human TNF-α were added together with *P. falciparum* sporozoites to fresh human primary hepatocytes. Medium was changed after 3 hr and every day after sporozoite inoculation. In both experimental settings, cultures were stopped 5 days later. Data are presented are the mean (± SD) reduction in liver schizont numbers in triplicate wells to the mean number in 6 control wells and are derived from one of two experiments. The numbers of *P. falciparum* 5 day-liver schizonts in the 6 control wells were 179.2±26.1. * *p*<0.05 versus control non-treated cultures (Kruskal-Wallis test, followed by Dunn test).

In order to determine how TNF-α inhibits liver stage parasite development, we used inhibitors that block the NO or the ROI pathways, both of which have been implicated previously in PE killing. First, we used S-methyl-thiourea (SMT), a competitive inhibitor of the inducible nitric oxide synthase [12]. Addition of SMT to cultures treated with 100 ng/ml of human TNF-α** did not reverse the effect of the cytokine against Pyy265BY HepG2/CD81 (Figure 5A) or against *P. falciparum* in primary human hepatocytes (Figure 6A). We next tested whether inhibition was mediated via the ROI pathway by using N-acetyl-cysteine (NAC), the precursor of glutathione and a potent endogenous antioxidant [13]. Addition of NAC did not reverse TNF-α mediated inhibition of Pyy265BY in HepG2/CD81 (Figure 5B), or that of *P. falciparum* in primary human hepatocytes (Figure 6A). We also observed that SMT and NAC did not reverse the inhibitory effect of TNF-α on PbA-infected HepG2-CD81 hepatoma cells (data not shown).

### A soluble mediator synthesized by human TNF-α-stimulated human hepatocytes prevents *P. falciparum* development

Since inhibition was observed only when cells were treated 48 h or 24 h before sporozoite infection but not on the time of sporozoite inoculation, we hypothesized that TNF-α-treated hepatocytes might release a parasite-inhibitory soluble mediator as previously shown for IL-1 or other inflammatory stimuli [28], [29]. *P. falciparum* sporozoites in normal medium were added to supernatant medium obtained from human hepatocytes treated for 48 h with TNF-α (1/1 volume). The mixture was then added to fresh human hepatocytes to initiate the infection. A strong significant inhibition of sporozoite development was observed, and it reached levels similar to those induced by direct TNF-α pre-treatment of the cultures (Figure 6B). This showed that an inhibitory soluble mediator was produced by TNF-α-stimulated hepatocytes.

## Discussion

The *in vitro* experiments presented here were designed to assess parameters, such as the origin of the cytokine, the origin of the host cells, the parasite species, or the schedule of application that might affect the anti-liver stage activity of TNF-α. Different types of hepatocytes and hepatoma cell lines together with 3 *Plasmodium* species were used. Our main finding is that human TNF-α was able to inhibit the hepatic development of two rodent malaria species, *P. yoelii* and to a lesser extent *P. berghei* (Figure 1), and most importantly that of *P. falciparum* (Figure 4). We showed that this activity of TNF-α was dependent on the host cell type and on the schedule of this cytokine's administration. Maximal inhibition could be obtained when human TNF-α was administered 48 h before sporozoite inoculation to HepG2-CD81 cells (Figure 1). Significant inhibition was still observed when cells were treated 24 h before sporozoite inoculation both in HepG2-CD81 infected with *P. yoelii* and in highly purified primary human hepatocytes infected with *P. falciparum*. The good concordance between the data derived from *P. yoelii*/HepG2-CD81 combination and that from the *P. falciparum*/human primary hepatocyte combination makes it a good surrogate in future studies of the role of non-specific immune components against the malaria hepatic stages. These results differed from those obtained using primary mouse hepatocytes where an effect was observed only when the cultures were treated 48 h (Figure 3) but not 24 h [11] before *P. yoelii* sporozoite inoculation. Of the different parameters that might account for this difference we favour those related to the TNF-αmechanism of action. Murine TNF-α and human TNF-α differ in their affinity to the various host TNF receptors. Human TNF-α signals only through TNFR1 in mouse cells [30] and, as shown here, it had no effect on primary mouse hepatocytes infected with *P. yoelii* or with *P. berghei* (Figure 3). Signalling by TNF-R1 signals is effected through the TRAD/NEMO pathway to NF-kB or through FAD to activate caspase for apoptosis. TNF-R2 also mediates NF-kB activation through the TRAF pathway [31], however, in hepatocytes only TNFR1 mediates activation of NF-kB [32]. The NF-kB pathway is necessary for the induction of the NO or ROI in hepatocytes [33]. These two mediators have been shown to inhibit the *Plasmodium* liver stage [34], [35]. We did not observe the induction or NO and ROI, which strongly suggests that it was TNFR2 but not TNFR1 that was involved in TNF-α signalling in infected hepatocytes. It has been proposed recently that malaria parasites manipulate their host hepatocytes to make them resistant to the apoptosis induced by TNF-α *in vivo* or *in vitro* [36] through interference with the NF-kB pathway [37] and consequently allowing them to escape the TNFR1-signaled cytotoxic effect of TNF-α. In addition, since signalling through TNF-R2 has also been involved in the necrotic effect of TNF-α [38], we tested whether treatment with TFN-α induced infected hepatocyte necrosis. Such an effect was ruled out because cell cytotoxicity was not observed in the MTT assays conducted after treatment with TNF-α.

The fact that the inhibitory effect of TNF-α was observed only when cultures were pre-incubated with the cytokine suggested that stimulated hepatocytes secrete an inhibitory factor and/or that the TNF-α treatment makes them refractory to infection. Host cell refractoriness is unlikely because addition of the supernatant from TNF-α-stimulated hepatocytes to the cultures was sufficient to obtain hepatic parasite inhibition. TNF-α alone or together with IL-6 and IL-1, is known to induce the synthesis of acute phase response proteins by hepatocytes. Although the acute phase response to inflammatory stimuli is evolutionary conserved, species-specific differences exist [39], [40]. IL-1 was previously shown to prevent sporozoite development in human or rat primary hepatocytes *in vitro* through the action of an acute phase protein, the C-reactive protein (CRP) [11], [29]. Human or rat C-reactive proteins can bind sporozoite and prevent their invasion and further development in hepatocytes [12], [30]. However, Yap *et al.* [41] have shown that CRP is not produced by human hepatocytes after TNF-α stimulation. They also showed that TNF-α treatment blocks the induction of CRP stimulated by IL-1 or IL-6 treatment of human hepatocytes. This suggest strongly that this acute phase protein does not mediate the TNF-α effect. It has been reported previously that two other acute phase proteins, the protease inhibitors α1-antitrypsin and α2 macroglobulin, were also able to prevent sporozoite infection and development [42]. Parasite proteases are necessary for sporozoite invasion in hepatocytes [43] and thus may be targeted by these two protease inhibitors. However, although TNF-α has been shown to increase the synthesis of α1-antitrypsin [44] or α2 macroglobulin [45] in HepG2 cells, it does not induce these molecules in human hepatocytes [40]. Thus, the nature of the inhibitory mediator secreted by human hepatocytes is still unknown and deserves further study. In the mouse liver the profile of acute phase proteins induced by inflammatory stimuli is different, for example mouse hepatocytes do not synthesize CRP. Serum Amyloid A is induced by TNF-α in mouse hepatocytes [46] and it might be responsible for the inhibition that is consequent to TNF-α-stimulation of mouse primary hepatocytes or mouse hepatoma cell lines. However, Serum Amyloid A is not induced in human hepatocytes by TNF-α stimulation [47] suggesting that other mediators might be involved.

During malaria blood stage infection, the production of TNF-α is increased [1], [3], and these cytokine might modulate new liver stage infections [48], [49]. By extension, any systemic inflammations or infections or more localized liver infections, of viral or bacterial origin, that induces high level of TNF-α might also have an inhibitory effect on the liver stages, which could consequently influence the outcome of a subsequent blood infection and its associated pathology [48], [49].
